# Differences in care burden, burnout, depression, quality of life and sleep quality between family members and professional caregivers of palliative care patients

**DOI:** 10.1186/s12904-026-02221-x

**Published:** 2026-07-07

**Authors:** Onur Aydoğdu, Yağmur Tetik-Aydoğdu, Özlem Oruç, H. Kerem Alptekin

**Affiliations:** 1 Department of Physiotherapy and Rehabilitation, Faculty of Health Sciences, Marmara University, Istanbul, Turkey; 2 Süreyyapaşa Chest Diseases and Thoracic Surgery Training and Research Hospital, Istanbul, Turkey; 3 Department of Physiotherapy and Rehabilitation, Bahçeşehir University, Istanbul, Turkey; 4 Kocaeli Health and Technology University, Kocaeli, Turkey

**Keywords:** Palliative Care, Caregivers, Burden, Family Members, Professional Support

## Abstract

**Background:**

The main aim of our study was to investigate whether there are differences in caregiver burden, burnout, depression, sleep quality and quality of life between family members and professional caregivers of palliative care patients. Furthermore, we examined the impact of burnout, depression, and sleep quality on the burden of care among primary caregivers of palliative care patients. Additionally, we investigated whether there were differences in these parameters among family members according to gender.

**Methods:**

This cross-sectional study involved a total of 134 individuals, including 67 palliative patients receiving care from the Palliative Care Service and 67 caregivers who provide primary care to these individuals. The outcome measurements for palliative care patients included falls efficacy, functional independence, quality of life, and cognitive function. For caregivers, the following variables were measured: caregiving burden, burnout, depression, sleep quality, and quality of life.

**Results:**

This study found that family members may experience a higher level of care burden, burnout, depression, sleep difficulties and lower quality of life compared to professional caregivers (*p* < 0.05). In addition, female family members exhibited worse scores for care burden, sleep quality and quality of life than male family members (*p* < 0.05). Increased caregiver burden in palliative care patients was associated with increased levels of burnout and depression, as well as sleep disturbance and reduced quality of life (*p* < 0.05).

**Conclusions:**

Our study suggests that providing primary caregivers of palliative care patients with support tailored to their specific needs in terms of burnout, care burden, depression, sleep and quality of life could be an important palliative care service.

## Introduction

The ageing of the population has resulted in an increase in the number of individuals requiring palliative care and in the number of individuals with chronic and serious illnesses who are remaining in palliative care for longer periods of time [1]. Palliative care is a patient-centred health service that aims to alleviate the severe suffering of patients and their families, thereby enabling them to have a better quality of life [2]. The involvement of family and professional caregivers in a palliative care approach by health professionals is a fundamental feature of palliative care [3]. Caregivers play an important role in providing palliative care by taking on practical tasks such as providing emotional support to the patient, relieving the patient’s pain or other symptoms, and communicating between the patient and healthcare professionals [1].

Caregivers provide a significant amount of care to palliative care patients, and this can have a negative impact on their health [4]. Caregivers can experience a significant burden when caring for a person with complex and serious illness, which can have a negative impact on their health and well-being [5]. It is recognised that caring is a burden for the caregivers [6]. Caregiving burden is defined as the distress experienced by caregivers as a result of providing care [7]. The presence of the caregiver is beneficial to the patient’s recovery process; however, the caregivers may experience negative conditions such as poor mental health, low quality of life, burnout or depression. These negative outcomes in the caregiver have been shown to be directly related to negative outcomes in the patient [8].

The greatest burden of caregiving falls on families, who are often traumatised, lack education and are unsupported [9]. In Western countries, it is estimated that between 70 and 90 per cent of long-term care is provided by a family caregiver [1]. A study reported that the majority of dementia patients receiving care at home were supported by their relatives [6]. In 2015, over 40 million family caregivers in the United States provided unpaid care to a parent, spouse, friend, or other adult loved one [10]. These caregivers are frequently the spouses or partners of patients, followed by children and in-laws. Most of these caregivers are women [6]. Although the majority of family caregivers are women, there has been a notable increase in the number of men taking on this role [10]. In some cases, professional caregivers are also involved [6]. The fact that care is provided by such a diverse range of individuals has led to a significant focus on the burden of caregiving in numerous studies. Conversely, the literature indicates a need for studies that compare the clinical outcomes of different types of caregivers.

In recent years, the literature has focused on several key areas of research concerning caregivers in palliative care. These include the estimation of the burden of care experienced by informal caregivers in palliative care [11], the identification of factors affecting the quality of life of caregivers of cancer patients in palliative care units [2], and the investigation of the burden of caregiving experienced by caregivers according to the functional level of elderly patients in oncological palliative care [12]. Additionally, studies have been conducted to examine the caregiving burden and quality of life experienced by family caregivers of palliative care patients [4]. Conversely, there is a lack of research comparing the differences in burden between informal and professional caregivers, despite this being an increasingly popular topic of investigation [6]. One of the few studies conducted for this purpose was on caregivers of patients with dementia. The results indicated that family caregivers exhibited higher scores on scales of caregiver burden compared to professional caregivers [6].

While many studies have focused on groups of caregivers providing primary care to patients with neurodegenerative diseases such as stroke, Parkinson’s disease, dementia, Alzheimer’s disease, etc., there is a need for data on caregivers in palliative care centres, where patients are in their final stages and where caregiving burden and burnout are likely to be highest. Furthermore, there is a need for studies that compare the burden of care between family members and professional caregivers of palliative care patients, particularly with regard to different caregiver-related parameters, or that examine differences by gender.

The main aim of our study was to investigate whether there are differences in caregiver burden, burnout, depression, sleep quality and quality of life between family members and professional caregivers of palliative care patients. Furthermore, Furthermore, we examined the impact of burnout, depression, and sleep quality on the burden of care among primary caregivers of palliative care patients. Additionally, we investigated whether there were differences in these parameters among family members according to gender.

## Methods

### Study design and setting

This was a cross-sectional study conducted in one of the public hospitals with the highest palliative care capacity located in Istanbul, Turkey.

### Study population

The study population comprised patients aged 65 and above who had been hospitalised in the palliative care unit for a minimum of one month. All of the geriatric patients included in the study were hospitalised in the palliative care service due to a variety of cancers, including those of the larynx, prostate, lung, respiratory system diseases (e.g., coronavirus disease, advanced chronic obstructive pulmonary disease, and pneumonia), neurological system diseases (e.g., Alzheimer’s disease, dementia, epilepsy, and stroke), or cardiologic system diseases (e.g., heart arrhythmia, hypertension, and heart failure).

The inclusion criteria were all individuals who provided primary care to geriatric patients in the Palliative Care Service for at least 1 month and volunteered to participate in the study. All carers were the primary caregivers, those who were most involved with the patients and spent the majority of their time with them.

In our study, the caregivers of geriatric patients were categorised as either family or professional caregivers. Family caregivers were defined as family members who provided care voluntarily and without financial compensation, while professional caregivers were defined as non-family members hired to provide care in exchange for wages as part of their professional occupation [13].

### Ethics

This research has obtained ethical approval from the Clinical Research Ethics Committe of Bahcesehir University (Protocol Number: 2021-07/02). The researcher has obtained written and signed informed consent or verbal permission from all of caregivers and geriatrics, where possible. Our study was conducted in accordance with the Declaration of Helsinki.

### Sampling method

The study sample size was calculated using the software G-Power version 3.1.9.3 (Heinrich Heine University Düsseldorf, Germany) and based on a study by Schreiner AS et al. assessing family caregiver’s mental health using a statistically derived cut-off score for the Zarit Burden Interview [14]. Schreiner AS et al. study was chosen as the reference study because it assesses the care burden on family caregivers of geriatric individuals with similar clinical characteristics to those in our study, and it uses the same outcome measurement tool (Zarit Caregiver Burden Scale). In this study, Zarit Burden Interview score as the burden levels of caregivers were calculated for the geriatric patients with general disabilities and for those with chronic obstructive pulmonary disease, 30.34 ± 17.7 and 20.35 ± 13, respectively. The calculation was based on an independent samples t-test. With 95% power and an α error coefficient of 0.05, at least 53 cases were required. In case of the possibility of interrupting the study, the number of cases for caregivers was increased by 25% to at least 67.

Palliative patients and their caregivers in the palliative care service were approached using non-probability convenience sampling. They were informed about the study and those who agreed to participate were screened for inclusion and exclusion criteria [11].

### Data collection tools

Data collection was carried out from July 2021 to July 2023 by a qualified physiotherapist with a PhD, who was in charge of the palliative care unit. Caregivers and geriatrics were asked to complete the survey questions themselves. If the older people were unconscious, their responses to socio-demographic information were obtained by caregivers and relatives. In addition, if requested by the geriatrics or caregivers, the physiotherapist read and marked the answers without any guidance. All face-to-face interviews were conducted in a quiet environment with sufficient time allowed.

### Outcome measures

In face-to-face interviews, sociodemographic information and physical characteristics including marital status, contact information (telephone numbers), and education level were questioned. For geriatrics, the number of falls in the last year and the relationship to the caregiver were recorded on the data collection form.

The outcome measurements for palliative care patients included falls efficacy, functional independence, quality of life, and cognitive function. For caregivers, the following variables were measured: caregiving burden, burnout, depression, sleep quality, and quality of life.

### For geriatrics

#### Falls efficacy

In this study, the Falls Efficacy Scale was used to assess the fear of falling experienced by all palliative patients when performing their daily activities. This is a 10-item scale consisting of bathing or showering, reaching for shelves, walking around the house, preparing meals, getting in and out of bed, answering the door or telephone, sitting in and out of a chair, dressing and undressing, personal care, going to and from the toilet. Individuals give a score between 1 (very confident) and 10 (not confident at all) for each question and all the scores are added up. If the total score is above 70, this indicates the presence of fear of falling. The Turkish validity and reliability study of the scale was conducted by Durmuş et al. in 2025 [15–17].

#### Functional independence

The Functional Independence Measurement Questionnaire was used to measure the level of independence of the subjects included in the study. The Functional Independence Measurement is an assessment tool that aims to measure the level of independence of the individual in basic physical and cognitive activities in daily life. The scale consists of 18 questions in total, each graded between 1 and 7 points. In the scale, level 1 is fully assisted, while level 7 indicates full independence. The higher the score, the higher the independence level of the individual [18].

#### Quality of daily life

Katz Index was used to measure the level of independence in activities of daily living. The scale is designed to assess the degree of independence an individual exhibits in performing activities of daily living. The six items, which comprise bathing, dressing, toileting, moving, continence and feeding, are graded using a binary scoring system (0 = dependent, 1 = independent). A score of six indicates that the individual is completely independent, whereas a score of zero indicates that the individual is completely dependent. The Turkish validity study of the scale was conducted by Arık et al. in 2015 [19].

#### Cognitive status

The cognitive function of the geriatric patients was evaluated using the Mini-Mental State Examination (MMSE) test. In the MMSE, which ranges from 0 to 30, a score of 24 and above indicates preserved cognitive function, while a score below 24 indicates cognitive impairment. The Turkish version of the scale was revised by Keskinoğlu et al. in 2009 and was developed as a version for use with older people living in both educated and uneducated communities [20–22].

### For caregivers

#### Caregiving burden

The Zarit Burden Interview was used to assess the caregiving burden in carers of all palliative geriatric individuals. The scale was developed by Zarit, Reever and Bach-Peterson in 1980. The scale is employed to evaluate the stress experienced by individuals assuming the role of caregivers for the elderly or individuals requiring care. The scale, which can be completed by the caregiver or the researcher, comprises 22 statements that assess the impact of caregiving on the individual’s life. The scale employs a Likert-type rating system, with responses ranging from 0 to 4, indicating the frequency of a particular behaviour or experience as follows: never, rarely, sometimes, frequently, often, or almost always. A Turkish validity and reliability study was conducted by F.H. İnci and M. Erdem. The scoring of the scale is conducted in the following manner: 0–20 points indicate a “no care burden,” 21–40 points indicate a “light care burden,” 41–60 points indicate a “moderate care burden,” and 61–88 points indicate a “heavy care burden.” [4, 23].

#### Burnout

The Burnout Scale-Short Form is the second most frequently employed measurement instrument in burnout research. The scale, which was developed by Pines and Aronson (1988) for use with all occupational groups, as well as non-professional groups, consists of twenty-one items and was prepared as a self-report on a seven-point scale for the evaluation of individuals’ physical, emotional and mental burnout levels. The Turkish adaptation, validity and reliability studies of the scale were carried out by Çapri in 2006 [24].

#### Depression

Beck Depression Inventory is a 21-item Likert-type self-report scale to measure the severity of depression. The participant is asked to rate symptoms in the ‘last week including today’. Each symptom is rated as absent, mild, moderate or severe. The total score ranges from 0 to 63. The validity and reliability study of the Turkish version of the scale was conducted by Dr Nesrin Hisli. Cronbach Alpha internal consistency coefficient was reported as 0.86 [25].

#### Sleep quality

The PSQI is a sleep questionnaire that helps to assess the quality of sleep, amount of sleep, presence and severity of sleep disturbances for the past month. The Turkish validity and reliability study was conducted by Ağargün et al. This scale has 19 items and measures seven subcomponents of sleep quality, including subjective sleep quality (C1), time to fall asleep (C2), sleep duration (C3), habitual sleep efficiency (C4), sleep disturbances (C5), use of sleep medication (C6) and daytime dysfunction (C7). The total PSQI score is the sum of the seven subscores and ranges from 0 to 21. The PSQI total score clearly distinguishes good sleepers (PSQI total score ≤ 5) from poor sleepers (PSQI > 5) [26].

#### Quality of life

The quality of life of caregivers was evaluated using the Short-Form 36 (SF-36). This scale, which has been extensively validated and is a widely used index for assessing various aspects of quality of life, comprises a total of 36 questions distributed across eight subscales: physical functioning, role physical, role emotional, vitality, mental health, social functioning, bodily pain, and general health. The total score for each subscale is calculated on a scale of 0 to 100, with higher scores indicating a greater quality of life [27, 28].

### Statistical analysis

All statistical analyses were performed using with the Statistical Package for the Social Sciences (SPSS) (IBM SPSS Statistics version 11.5, Chicago, IL, USA). The mean and standard deviation were calculated as descriptive statistics for each measure and for characteristics of palliative care patients and caregivers. All statistical tests were two-tailed and the statistical significance level was set at *p* < 0.05. The Shapiro-Wilk test was used to determine whether the data followed a normal curve distribution. Given that the data did not conform to a normal distribution, non-parametric analyses were conducted. Grubb’s test was used to check whether outliers were present at an alpha of 0.05. In the case of finding an outlier, that value was removed from the dataset. Pairwise deletion of missing data was used, and data were only excluded from calculations where no score was available. The Mann-Whitney U test was used to examine mean differences between family and professional caregivers. In addition, the Mann-Whitney U test was used to analyse whether there was a difference between male and female caregivers in terms of the relevant parameters. Effect sizes (Cohen’s d) were calculated to determine the magnitude of the difference between the groups. The Cohen’s d results are as follows: 0.8 for large effects, 0.5 for medium effects and 0.2 for small effects [29].

The relationship between caregiver burden, burnout, depression, sleep quality and quality of life among caregivers was examined using the Spearmen Correlation Test. In order to classify the correlations, the following criteria were considered: trivial (0–0.1), small (0.1–0.3), moderate (0.3–0.5), large (0.5–0.7), very large (0.7–0.9), nearly perfect (0.9), and perfect (1.0) [30].

Multiple linear regression analysis was used for the variables predicting the caregiving burden scores.

## Results

### Participants

We recruited 67 palliative care patients (53.7% female; 79.35 ± 7.95 average of years) and 67 primary caregivers (76.1% female; 51.95 ± 11.16 average of years) in this study. Table 1 presents the sociodemographic and physical characteristics data for palliative care patients and their caregivers.

**Table 1:** Sociodemographic and physical characteristics of geriatrics and caregivers

Characteristic	Geriatrics (M ± SD, *n* (%))	Caregiver (M ± SD, *n* (%))
Age (years)	79.35 ± 7.95	51.95 ± 11.16
Body Height (cm)	164.82 ± 9.75	164.89 ± 8.27
Body Weight (kg)	64.93 ± 13.98	76.38 ± 14.40
BMI (kg/m^2^)	23.87 ± 4.83	28.10 ± 4.96
Gender		
Female	36 (53.7%)	51 (76.1%)
Male	31 (46.3%)	16 (23.9%)
Marital Status		
Married	30 (48.4%)	48 (77.4%)
Widow/Divorced	31 (50.0%)	7 (11.3%)
Single/Separated	1 (1.6%)	7 (11.3%)
Missing/Not Stated	5	5
Education Level		
Illiterate	21 (%32.3)	1 (%1.6)
Primary School	34 (%52.3)	26 (%40.6)
Elemantary	1 (%1.5)	8 (%12.5)
High-School	3 (%4.6)	8 (%12.5)
University	6 (%9.2)	21 (%32.8)
Missing/Not Stated	2	3

*M* Mean, *SD* Standard deviation, *n* number, *%* percentage, *BMI* Body mass index

It was found that 73.1% of the palliative patient group in whom the caregivers provided care were “family members”. Of 67 palliative care patients with a Mini-Mental State Examination score of 6.07 ± 9.13, 38 (56.7%) reported that they had never had a fall in the past year. The caregiving burden score for caregivers with a burnout score of 2.77 ± 1.43 was 28.84 ± 17.86 according to the Zarit Burden Index. In our study, the vast majority of professional caregivers were people who had previously provided care on many occasions, but who were not healthcare professionals. The majority of the family caregivers were typically the geriatric patient’s own daughter and had almost no experience of caring for anyone. Table 2 details the complete outcome variables of caregivers and palliative care patients.

**Table 2:** Variables of caregivers and palliative care patients

Geriatrics		Variables		Caregiver	
(M ± SD, *n* (%))					(M ± SD, *n*)
85.13 ± 27.51	Falls Efficacy Scale	Falls Eficacy	Caregiving Burden	Zarit Burden Index	28.84 ± 17.86
25.02 ± 23.28	FIM-Motor	Functional Independence Level	Burnout	Burnout Index	2.77 ± 1.43
12.70 ± 11.37	FIM-Cognitive		Depression	Beck Depression	12.70 ± 10.82
37.31 ± 32.73	FIM-Total		Sleep Quality	Pittsburgh	7.69 ± 3.65
0.74 ± 1.68	Katz Scale	Quality of Daily Life	Quality of Life / SF-36	Physical Functioning	83.84 ± 18.38
6.07 ± 9.13	Mini Mental State Examination	Cognitive Function		Role Physical	40.61 ± 44.82
38 (56.7%)	Never	Falls Number at last year		Role Emotional	45.63 ± 46.98
11 (16.4%)	1–2			Vitality	45.92 ± 27.52
18 (26.9%)	3 and more			Mental Health	58.95 ± 23.82
49 (73.1%)	Family Caregivers	Relationship to Caregiver		Social Functioning	54.42 ± 32.73
18 (26.9%)	Professional Caregivers			Bodily Pain	62.26 ± 27.34
				General Health	61.84 ± 19.07

M Mean, *SD* Standard deviation, *n* number, % Percentage, *FIM* Functional Independence Measure, *SF-36 *Short Form-36

### Comparison of variables between family members and professional caregivers, and between family members by gender

Table 3 reflects the comparison of variables between professional caregivers and family members. Statistically significant differences were found between family members and professional caregivers in terms of caregiving burden (*p* = 0.001), burnout (*p* = 0.044), depression (*p* = 0.001), sleep quality (*p* = 0.013), and quality of life (*p* < 0.05). In addition, statistically significant differences were found in terms of caregiver burden (*p* = 0.029), sleep quality (*p* = 0.035), and quality of life scores (*p* < 0.05) among family caregivers by gender.

**Table 3:** Comparison of gender-related differences in variables among family caregivers and comparison of variables between professional caregivers and family members

	Family Caregivers				All Caregivers			
Variables	Female Family Caregivers(Mean ± SD)*n* = 34	Male Family Caregivers(Mean ± SD)*n* = 15	*p*	Cohen’s d	Family Caregivers(Mean ± SD)*n* = 49	Professional Caregivers(Mean ± SD)*n* = 18	*p*	Cohen’s d
Caregiving Burden	37.75 ± 16.12	26.73 ± 16.10	0.029*	0.684	34.23 ± 16.76	14.77 ± 12.30	0.001*	1.323
Burnout	3.24 ± 1.46	2.49 ± 1.55	0.085	0.498	3.01 ± 1.51	2.17 ± 1.00	0.044*	0.655
Depression	17.06 ± 11.17	11.26 ± 9.26	0.129	0.565	15.21 ± 10.85	6.16 ± 7.73	0.001*	0.960
Sleep Quality	9.12 ± 3.55	6.80 ± 3.50	0.035*	0.658	8.38 ± 3.66	5.88 ± 3.00	0.013*	0.747
Physical Functioning	78.90 ± 20.50	89.00 ± 10.21	0.101	0.623	82.12 ± 18.37	88.33 ± 18.14	0.028*	0.340
Role Physical	17.96 ± 35.48	41.66 ± 44.98	0.038*	0.585	25.53 ± 39.86	80.00 ± 31.48	0.001*	1.516
Role Emotional	20.82 ± 38.56	51.10 ± 50.18	0.036*	0.676	30.49 ± 44.40	85.17 ± 26.14	0.001*	1.500
Vitality	32.03 ± 23.48	53.33 ± 30.03	0.018*	0.790	38.82 ± 27.33	64.44 ± 18.14	0.001*	1.104
Mental Health	47.75 ± 22.69	72.00 ± 20.50	0.001*	1.121	55.48 ± 24.60	68.00 ± 19.45	0.074	0.564
Social Functioning	40.23 ± 28.87	53.33 ± 34.86	0.165	0.409	44.41 ± 31.14	80.55 ± 20.21	0.001*	1.376
Bodily Pain	54.21 ± 25.88	68.00 ± 28.53	0.006*	0.506	58.61 ± 27.23	71.80 ± 25.99	0.083	0.495
General Health	55.15 ± 19.07	69.33 ± 17.40	0.023*	0.776	59.68 ± 19.54	67.50 ± 16.99	0.156	0.427

Mann Whitney U Test, *p* < 0.05*

### Relationships between variables

There were large-positive correlations between caregiving burden and burnout (*r* = 0.640, *p* = 0.001), depression (*r* = 0.533, *p* = 0.001), sleep quality (*r* = 0.575, *p* = 0.001), and quality of life (*p* < 0.05) in caregivers. A detailed examination of these values can be found in Table 4.

**Table 4:** Correlation matrix of study variables for caregivers

Variables	Correlation Coefficients	Variables				Quality of Life							
		1	2	3	4	Physical Functioning	Role Physical	Role Emotional	Vitality	Mental Health	Social Functioning	Bodily Pain	General Health
1.Caregiving Burden	r	-	0.640	0.533	0.575	-0.330	-0.649	-0.678	-0.637	-0.595	-0.673	-0.516	-0.571
	p		0.001*	0.001*	0.001*	0.007*	0.001*	0.001*	0.001*	0.001*	0.001*	0.001*	0.001*
2.Burnout	r	0.640	-	0.713	0.537	-0.339	-0.588	-0.595	-0.736	-0.711	-0.606	-0.524	-0.729
	p	0.001*		0.001*	0.001*	0.006*	0.001*	0.001*	0.001*	0.001*	0.001*	0.001*	0.001*
3.Depression	r	0.533	0.713	-	0.587	-0.358	-0.646	-0.637	-0.800	-0.701	-0.688	-0.471	-0.647
	p	0.001*	0.001*		0.001*	0.003*	0.001*	0.001*	0.001*	0.001*	0.001*	0.001*	0.001*
4.Sleep Quality	r	0.575	0.537	0.587	-	-0.366	-0.626	-0.611	-0.572	-0.646	-0.527	-0.501	-0.516
	p	0.001*	0.001*	0.001*		0.003*	0.001*	0.001*	0.001*	0.001*	0.001*	0.001*	0.001*

Spearmen Correlation Test; *p < 0.05

There were no significant correlations between age, body mass index, level of cognition and functional independence, and independence in activities of daily living of geriatric patients and caregiver burden, depression and burnout of palliative caregivers (*p* > 0.05).

There were significant correlations between the number of falls experienced by palliative patients over the past year and the levels of caregiver burden (*r* = 0.277, *p* = 0.028), burnout (*r* = 0.442, *p* = 0.001), and depression (*r* = 0.292, *p* = 0.020). Furthermore, there were statistical relationships between the age (*r*=-0.245, *p* = 0.049) and body mass index (*r*=-0.325, *p* = 0.011) of palliative patients and the sleep quality of their caregivers.

### Regression analysis for the prediction of caregiving burden scores by burnout, depression, and sleep quality

Table 5 shows the results of the regression analysis for the prediction of caregiving burden scores by burnout, depression, and sleep quality. The variables of burnout, depression, and sleep quality showed a significant relationship with caregiving burden (*R* = 0.699, R^2^ = 0.464, *p* < 0.001). Burnout, depression, and sleep quality variables explained 46.4% of the total variance in caregiving burden.

**Table 5:** Regression analysis of the prediction of caregiving burden scores according to burnout, depression, and sleep quality

Variables	B (95% CI)	SE	β	t	*p*
Constant	1.595	4.379		0.364	0.717
Burnout	5.251	1.609	0.420	3.264	0.002*
Depression	0.197	0.226	0.119	0.873	0.386
Sleep Quality	1.350	0.559	0.277	2.415	0.019*

R = 0.699, Adj.R^2^ = 0.464, F = 19.150, *p* = 0.001

*Adj.R*^2^Adjusted R square, *SE* Standart Error

*p < 0.05

According to the standardized regression coefficient (β), burnout, depression, and sleep quality variables were found to be significant predictors of caregiving burden. According to the results of the t-test on the significance of the regression coefficients, the burnout and sleep quality levels were significant predictors of caregiving burden of caregivers.

## Discussion

The primary objective of our study was to investigate whether there were differences between family members and professional caregivers of palliative patients in terms of care burden, burnout, depression, sleep quality and quality of life. In addition, we hypothesised that there might be differences in variables by gender among family members who provide care to palliative patients as their primary carers.

The main finding of our study was that family members reported worse caregiving burden, burnout, depression, sleep and quality of life than professional caregivers. Given the evidence from the majority of studies that younger age, female gender, lower educational status, unemployment, poor health, and especially being a relative of the patient are significant predictors of reduced quality of life in the caregiver [31], this finding was to be expected. Furthermore, family members may perceive a greater burden due to the cognitive impairment of people with dementia, as well as aggressive attitudes and behaviours, in comparison to professional caregivers [6]. This may be due to the fact that family members often have insufficient practical knowledge. For this reason, research has highlighted the importance of effective communication and information sharing between patients, carers and healthcare professionals [5].

A study by Seidel and Thyrian, similar to our study, reported that family members had higher scores on depression and caregiver burden scales compared to professional caregivers. On the other hand, the same study indicated that the quality of life scores of professional caregivers were lower than those of the control group [6]. In our study, quality of life scores were higher in professional caregivers, as were other parameters. Family members with limited training in providing care are more likely to have lower QoL scores because they helped with activities of daily living and personal tasks such as bathing, dressing, wound care, arranging transport, managing medication and finances [10].

The study revealed significant differences in care burden, sleep quality and quality of life among family members according to gender. Female family members exhibited worse scores for care burden, sleep quality and quality of life than male family members. This finding was in accordance with the hypothesis. As with our own findings, the study conducted by Seidel and Thyrian revealed that female family carers reported higher levels of care burden than their male counterparts [6]. The study by Rezende et al. reported that the highest rates of care burden were observed among female carers [12]. Typically in societies, boys are taught to be dominant and not to show emotional vulnerability, whereas girls are encouraged to be more sensitive to the needs of others through empathy [10]. This supports the aforementioned finding. Previous studies and existing data show that among the carers who provide care to palliative care patients, women have a higher burden of care [12].

The emotional responses of caregivers have been shown to impact the quality of their lives [31]. Given that women tend to experience more intense emotional reactions than men, it is to be expected that there would be differences between the genders in terms of these parameters. However, our study did not reveal any significant gender differences in the prevalence of burnout and depression among family members. We believe that this is likely due to the fact that these conditions tend to develop and manifest over an extended period of time.

Another noteworthy finding was that an elevated care burden on caregivers of palliative patients was associated with increased burnout and depression levels, as well as impaired sleep and a diminished quality of life. The findings of this research are consistent with the results observed in previous studies. For instance, Cengiz et al. [4] demonstrated that there is a significant relationship between the caregiver’s quality of life and their care burden. Additionally, other studies have indicated that an increase in care burden can lead to a deterioration in the caregiver’s general well-being [4, 32, 33]. The results of the study by Rha et al. show that the burden of care is one of the most powerful factors affecting quality of life [7]. In light of these findings, the strong association between caregiver burden and caregiver burnout, sleep and quality of life suggests that interventions to reduce caregiver burden should be supported wherever possible.

To the best of our knowledge, there are no studies in the existing literature that focus on the sleep quality levels of carers. However, it is possible to observe a deterioration in sleep quality over a relatively short period of time when the quality of life of carers is adversely affected by an increase in their care burden and burnout levels.

The study findings indicated that the age, body mass index, cognitive level, functional independence and independence in activities of daily living of palliative patients were not associated with caregiving burden, burnout and depression levels in carers. On the other hand, current research demonstrated that as the frequency of falls among palliative patients in the last year increases, the levels of caregiving burden, burnout, and depression in caregivers also escalate. In addition, it is noteworthy that as the age and body mass index of palliative patients increased, the quality of sleep experienced by their caregivers also improved. The level of caregiver burden may vary according to a number of clinical and sociodemographic characteristics of palliative patients. This may subsequently affect other health-related parameters of caregivers, such as quality of life [31]. It has been demonstrated that the burden experienced by carers increases in line with the functional decline of the patient. The results of a study indicated that there were significant correlations between the functional status of the patient and the caregiving burden, as well as the quality of life of the carer [7]. On the other hand, the results of the current study indicated that not all clinical and functional findings of palliative patients may directly increase the burden on caregivers. It was therefore considered that the number of falls experienced by patients might be a significant factor in this regard. The correlation between the age and body mass index of palliative patients and the sleep quality of caregivers can be attributed to the fact that as the patient’s level of bed dependency increases, the demands on the caregivers may decrease and subsequently improve their sleep quality.

It is additionally worth noting that the present study identified burnout and sleep quality as significant predictors of caregiving burden among caregivers. As a result, it was determined that having higher burden and sleep quality scores predicted higher caregiving burden scores in the caregivers. One study found that caregiver burden explained a significant proportion of the variance in quality of life scores [7]. Another study found that low functional status was the strongest predictor of caregiver burden [34]. In conclusion, although burnout and sleep quality were important predictors of caregiver burden in our study, they should not be considered as sole indicators. Therefore, it is recommended that caregivers with good burnout and sleep quality should also be assessed for caregiver burden.

Although not one of the primary objectives of this study and not measured using subjective scales, certain assessment parameters, such as caregiving intensity, cohabitation status and socioeconomic status, have the potential to influence outcomes relating to burden, burnout, depression, quality of life and sleep quality. Schulz et al. have noted that caregiving intensity (the number of hours spent on care per week), family ties (particularly with spouses), and cohabitation with geriatric individuals are strong predictors of adverse psychological outcomes [35, 36]. An increase in caregiving intensity or living with a geriatric individual involves spending more time with them. This may lead to greater stress and more intense emotional experiences. Lower socioeconomic status has been shown to be associated with an increased burden on the caregivers [37]. Another study found that the higher an individual’s socioeconomic status, the lower the likelihood of the caregiver experiencing depression [38]. In this study, these parameters could not be obtained from every patient in a standardised manner; therefore, they could not be presented as findings. However, it was considered that parameters such as caregiving intensity, cohabitation status and socioeconomic status should be regularly assessed in caregivers using standardised methods.

### Limitations and strength

Our study has some limitations. While the study data were obtained from one of Istanbul’s largest hospitals with regard to palliative care, this represents a limitation in terms of the generalisability of the results to the general population. Secondly, the study did not examine the discrepancies between the experience levels of the professional carers included in the sample. In addition, information on the caregivers’ prior work experience, wages, living conditions, employment status and certified training in this field is unavailable. Furthermore, the number of professional and family carers was not distributed in a balanced manner. Last but not least, all evaluations were conducted in a single session. Given the cross-sectional nature of the study, it is probable that all responses were influenced by the emotional state of the carers and palliative patients on the day in question.

To the best of our knowledge, this is the first study which aims at describing family members’ and professional caregivers’ burden, burnout, depression, life and sleep quality after hospitalization of the palliative patients. In view of the findings of this study, it is recommended that patient-centred outcomes among caregivers of palliative geriatric patients receiving palliative care be monitored in future multicentre, longitudinal, randomised controlled trials following the implementation of specific treatment approaches (such as exercise-based approaches, mindfulness-based approaches, anxiety, stress and depression management, sleep training, and coping with social isolation). In addition, research could be conducted among carers of geriatric individuals requiring palliative care, focusing on specific subgroups (such as those with oncological causes like lung cancer, infections, neurological diseases, advanced respiratory problems, etc.), utilising technology-supported care, digital interventions and group therapy.

## Conclusion and recommendations

1. Our study revealed that family members may experience a higher level of care burden, burnout, depression, sleep difficulties and lower quality of life compared to professional caregivers.
2. Female family members exhibited worse scores for care burden, sleep quality and quality of life than male family members.
3. Increased caregiver burden in palliative care patients was associated with increased levels of burnout and depression, as well as sleep disturbance and reduced quality of life.
4. In this cross-sectional study, it was found burnout and sleep quality to be significant predictors of caregiving burden. However, causal relationships cannot be inferred.

Supporting people who continue to care for patients who are hospitalised in palliative care can be indirectly considered as a palliative care service. It may therefore be possible to improve the quality of palliative care by identifying various problems affecting different types of carers.

## Data Availability

The data that support the findings of this study are available on reasonable request from the corresponding author.
